# Early Life BMI Trajectories and Associations With Childbirth and Infertility Among Women

**DOI:** 10.1001/jamanetworkopen.2026.26893

**Published:** 2026-08-04

**Authors:** Dorthe C. Pedersen, Julie Aarestrup, Lise G. Bjerregaard, Elisabeth W. Andersen, Allan Jensen, Susanne K. Kjær, Petur B. Juliusson, Kathleen M. Rasmussen, Jennifer L. Baker

**Affiliations:** 1 Center for Clinical Research and Prevention, Copenhagen University Hospital–Bispebjerg and Frederiksberg, Copenhagen, Denmark; 2 Virus, Lifestyle and Genes, Danish Cancer Institute, Copenhagen, Denmark; 3 Department of Gynecology, Copenhagen University Hospital, Rigshospitalet, Copenhagen, Denmark; 4 Department of Health Registry Research and Development, National Institute of Public Health, Bergen, Norway; 5 Department of Clinical Science, University of Bergen, Bergen, Norway; 6 Division of Nutritional Sciences, Cornell University, Ithaca, New York

## Abstract

**Importance:**

Concomitant with the global obesity epidemic, fertility has declined in Western countries with birth rates falling below replacement levels. A higher body mass index (BMI) among women is associated with reduced fertility, but associations with body size earlier in life are underexplored.

**Objective:**

To investigate whether trajectories of childhood BMI are associated with timing of first childbirth and infertility in women.

**Design, Setting, and Participants:**

This prospective, population-based cohort study used linked national register and school health examination data of women aged 18 to 45 years who were born in Copenhagen, Denmark, between 1950 and 1989. Participants were followed up for reproductive outcomes from January 1, 1977 to December 31, 2022. Analytic work was conducted from May to October 2025.

**Exposure:**

Measured height and weight data used to calculate childhood BMI trajectories from ages 6 to 15 years.

**Main Outcomes and Measures:**

Registration of childbirth and hospital-based diagnoses of infertility identified in the Medical Birth Register, the Danish Infertility Cohort, the National Patient Register, and the In Vitro Fertilization Register. Hazard ratios (HRs) and 95% CIs were estimated using Cox regression models.

**Results:**

This study included 60 864 women in the childbirth analysis, of whom 47 669 (78.3%) gave birth (median [IQR] age, 27.2 [23.7-30.9] years); 58 148 were included in the primary infertility analysis, of whom 5381 (9.3%) were diagnosed with primary infertility (median [IQR] age, 31.3 [27.9-35.1] years); and 63 066 were included in the any infertility analysis, of whom 6961 (11.0%) were diagnosed with any infertility (median [IQR] age, 31.9 [28.4-35.7] years). Women with an obesity trajectory in childhood had a lower hazard of giving birth than those with an average trajectory, and this hazard decreased with increasing age (ages 25-29 years: HR, 0.78 [95% CI, 0.72-0.86]; ages 35-45 years: HR, 0.56 [95% CI, 0.46-0.67]). Similarly, women with an overweight trajectory during childhood had a lower hazard of giving birth, which decreased with increasing age. No associations were observed between the childhood BMI trajectories and primary or any infertility.

**Conclusions and Relevance:**

In this cohort study of Danish women, associations with childbirth decreased from mid- to late-reproductive ages, when the window of fertility narrows. These findings suggest that childhood body size is associated with timing of childbirth, highlighting the importance of addressing women’s reproductive health early in the life course.

## Key Points

**Question:**

Are childhood body mass index (BMI) trajectories associated with timing of first childbirth and infertility in women?

**Findings:**

In this cohort study of 60 864 women followed for childbirth, an obesity trajectory during childhood was significantly associated with a lower hazard of childbirth; this hazard decreased with advancing reproductive ages. Among women followed for primary infertility (n = 58 148) and any infertility (n = 63 066), childhood BMI trajectories were not associated with these outcomes.

**Meaning:**

These findings suggest that childhood body size may be associated with later reproductive outcomes in women, highlighting the importance of a life-course approach to investigate women’s reproductive health.

## Teaser

This cohort study examines whether childhood body mass index (BMI) is associated with reproductive outcomes later in life among women in Denmark.

## Introduction

Globally, fertility rates are decreasing. In 2021, the estimated number of children born per woman during her reproductive life was 2.2 compared with 4.8 in 1950.^1^ In many parts of the world, total fertility rates are below the 2.1 threshold required for population replacement.^1^ In high-income countries, the percentage of women who remain childless has risen, with rates in the US increasing from 10% among women born in 1945 to approximately 15% among those born in 1970.^2^ Nevertheless, it has been estimated that only about 5% of European women aged 18 to 40 years do not intend to have children.^3^ Thus, most childlessness is likely involuntary. Adding to the fertility crisis, infertility, defined as having inability to conceive after 12 months of regular, unprotected sexual intercourse,^4^ now affects nearly 7.5 million European women of reproductive age.^5^

Although advancing age is a major contributor to childlessness and infertility, overweight and obesity further increase these risks through physiological and social pathways.^6,7^ In the US and Europe, 1 in 3 school-aged girls now has overweight or obesity,^8,9^ suggesting they are likely to enter their reproductive years with excess weight. However, little is known about whether childhood body mass index (BMI) is associated with childlessness and infertility in adulthood. Findings from the few available studies in this area are inconsistent; 2 studies suggest that overweight and obesity in childhood increase risk of childlessness in adulthood,^10,11^ 2 report no associations with infertility,^11,12^ and 2 indicate an increased risk of infertility.^13,14^ Because declining fertility may have adverse societal consequences,^1,15^ further evidence on whether childhood body size relates to risk of childlessness and infertility is needed to guide social and health policies. Therefore, we investigated whether trajectories of BMI across childhood were associated with childbirth and infertility in adulthood in a large cohort of Danish women. We hypothesized that the higher the childhood BMI, the higher the risks of infertility and not giving birth.

## Methods

### Data Linkages and Study Population

Data linkages for this prospective, population-based cohort study were approved by the Danish Data Protection Agency. The data collection for the Danish Infertility Cohort was approved by the Danish Health Data Board and Statistics Denmark and the Local Ethical Committees for Copenhagen and Frederiksberg municipalities and approved and registered in the archive list of the Danish Cancer Institute. According to Danish legislation, ethical approval or written consent is not required for register-based research. We followed the Strengthening the Reporting of Observational Studies in Epidemiology (STROBE) reporting guideline.

Women for this study came from the Copenhagen School Health Records Register, a registry of nearly every child born between 1930 and 1996 who ever attended a private or public school in the Copenhagen municipality of Denmark.^16^ Children underwent legally mandated health examinations from ages 6 to 15 years, in which trained school clinicians measured the child’s height and weight following standardized procedures.^16^

On April 2, 1968, all Danish citizens who were alive and living in Denmark were given a unique identification number, which is used in all national registers.^17^ The identification number is available for approximately 90% of the individuals in the cohort.^16^ Using this number for individual-level linkage, we obtained information on migration and vital status from the Civil Registration System (CRS).^17^ Women who gave birth were identified in the Medical Birth Register, which includes pregnancies ending in a live birth or stillbirth since 1973.^18^ We included both live births and stillbirths. Information on infertility was retrieved from 3 resources. The Danish Infertility Cohort includes all women with infertility who were referred to a private clinic or a public hospital between 1963 and 1998.^19^ The National Patient Register (NPR) holds information on all inpatient hospital contacts since 1977 and outpatient clinic contacts since 1995.^20^ We classified infertility diagnoses using the *International Classification of Disease, Eighth Revision* (code 628) until 1994, and thereafter, the *International Classification of Disease and Other Related Health Problems, Tenth Revision* (code N97). The In-Vitro Fertilization (IVF) Register includes comprehensive data from all public and private fertility clinics on intracytoplasmic sperm injection, frozen embryo replacements, vitrified-warmed blastocyst replacement, and oocyte donation since 1994, and intrauterine insemination procedures since 2006.^21^ *Primary infertility* was defined as an infertility diagnosis while nulliparous. *Any infertility* was defined as an infertility diagnosis regardless of childbirth history. Information on operations for bilateral oophorectomy, hysterectomy, or tubal ligation came from the NPR.

Eligible women for this study had a valid identification number and were born in 1950 or later (eFigure in Supplement 1). For the childbirth analyses, women had to reside in Denmark on January 1, 1977, or on their 18th birthday, whichever came last; for analyses on infertility, the corresponding age was 20 years. Follow-up began at different ages because childbirth can occur earlier than an infertility diagnosis. We excluded women with a bilateral oophorectomy, hysterectomy, or tubal ligation; those who had given birth or been diagnosed with infertility before study entry; and those missing information on BMI trajectories and parental education (eFigure in Supplement 1). Follow-up ended at the event of interest; age 45 years; a bilateral oophorectomy, hysterectomy, or tubal ligation; death; migration; loss to follow-up; or December 31, 2022, whichever occurred first. Additionally, for analyses of primary infertility, women who gave birth before study entry were excluded, and after study entry censored on the date of childbirth. More details the of study populations can be found in the eMethods in Supplement 1.

### Childhood BMI

Childhood BMI was calculated as weight in kilograms divided by height in meters squared using recorded weight and height. Childhood BMI trajectories were identified using latent class trajectory models, as previously described.^22^ Girls with at least 2 BMI values were included; 90% of girls had 3 to 10 values. Trajectories were modeled using natural splines to capture growth changes. Based on model fit indices and visual inspection, a 5-class model without random effects was selected, yielding noncrossing trajectories labeled below average, average, above average, overweight, and obesity.^22^ Posterior probabilities of trajectory membership were calculated for each individual. Further description can be found in the eMethods in Supplement 1. For secondary analyses, we further classified BMI values at the single ages 7 and 13 years to reflect ages before and during puberty. We used the US Centers for Disease Control and Prevention’s age- and sex-specific BMI percentiles for underweight (*<*5th), normal weight (5th to <85th), overweight (85th to <95th), and obesity (≥95th),^23^ because this reference is widely used internationally.

### Covariates

As a proxy for childhood socioeconomic position (SEP), we used information on the highest completed parental education from the Educational Attainment Register.^24^ Educational attainment was categorized into low (less than or equal to lower secondary), medium (upper or postsecondary), and high (tertiary). Civil status, obtained from the CRS, was classified as ever or never married before age 45 years.

### Statistical Analysis

The data were analyzed from May to October 2025. Characteristics of the women are presented as medians and IQRs for continuous variables and frequencies (percentages) for categorical variables by assigned childhood BMI trajectory (trajectory with the highest posterior probability). In our primary analyses, we estimated cause-specific hazard ratios (HRs) and 95% CIs using Cox proportional hazard models for associations between childhood BMI trajectories and childbirth, primary infertility, and any infertility, respectively, using the average BMI trajectory as the reference. The posterior probability of belonging to each childhood BMI trajectory was used as the exposure to minimize risks of misclassification.^25^ The HRs should be interpreted as the change in risk associated with a higher posterior probability of belonging to a given trajectory relative to the other trajectories. Age was the underlying timescale, and models were stratified by 10-year birth cohorts (1950-1959, …, 1980-1989) to allow cohort-specific baseline hazards. Adjusted models additionally included information on parental education.

To evaluate proportional hazards assumptions, the time scale was split into 4 age groups (<25, 25-29, 30-34, 35-45 years) to reflect established reproductive life stages and clinical thresholds. We used likelihood ratio tests to examine whether associations differed across the age groups. Because violations of the assumption for associations with childbirth were detected (*P* ≤ .001), the age-specific hazards ratios are presented. No violations were detected for associations with primary or any infertility. Potential interactions between childhood BMI trajectories and birth cohorts for associations with primary and any infertility were tested using likelihood ratio tests, but none were detected.

In secondary analyses, we analyzed categories of BMI at ages 7 and 13 years as exposures to facilitate comparison with previous research. Additionally, in sensitivity analyses, we analyzed associations between childhood BMI trajectories and childbirth among ever-married women to minimize social confounding related to partnering (eMethods in Supplement 1). Information on male infertility (*ICD-10* code N974) became available from 1994 in the NPR and IVF Register, and information on same-sex partnership and single status became available from 2006 in the IVF Register. To account for IVF treatments unrelated to female infertility, women with these registrations were censored in the infertility analyses. In other analyses of primary infertility, we initiated follow-up at age 30 years or older, which is the age when most women in Denmark start seeking treatment for infertility. All analyses were performed in Stata, version 19.0 (StataCorp LLC). A 2-sided *P* < .05 was considered statistically significant.

## Results

Of the 84 508 women in the starting population (eFigure in Supplement 1), we included 60 864 women in the childbirth analyses, of whom 47 669 (78.3%) gave birth at a median (IQR) age of 27.2 (23.7-30.9) years; 58 148 women in the primary infertility analyses, of whom 5381 (9.3%) were diagnosed at a median (IQR) age of 31.3 (27.9-35.1) years; and 63 066 women in the any infertility analyses, of whom 6961 (11.0%) were diagnosed at a median (IQR) age of 31.9 (28.4-35.7) years (Table 1). In all 3 analytic study populations, most women had an average BMI trajectory during childhood, whereas only 3.8% had an obesity BMI trajectory. Moreover, women with an overweight or obesity BMI trajectory in childhood were born in later birth cohorts than women with an average BMI trajectory (Table 1).

**Table 1.** Characteristics by Childhood BMI Trajectory of the Included Women From the Copenhagen School Health Records Register

Characteristic	Total	Childhood BMI trajectory				
		Below average	Average	Above average	Overweight	Obesity
**Childbirth**						
No. of women (%)^a^	60 864 (100)	13 460 (22.1)	22 608 (37.1)	15 431 (25.4)	7060 (11.6)	2305 (3.8)
Year of birth, median (IQR), y	1969 (1961-1979)	1968 (1960-1978)	1968 (1961-1977)	1969 (1961-1979)	1971 (1963-1981)	1978 (1966-1985)
Women with childbirth, No. (%)	47 669 (78.3)	10 617 (78.9)	18 062 (79.9)	12 082 (78.3)	5319 (75.3)	1589 (68.9)
Age when giving birth, median (IQR), y	27.2 (23.7-30.9)	27.4 (23.9-31.0)	27.3 (23.8-31.0)	27.1 (23.6-30.7)	26.9 (23.4-30.7)	26.6 (23.1-30.4)
Parental educational level, No. (%)						
High	17 163 (28.2)	3846 (28.6)	6592 (29.2)	4397 (28.5)	1836 (26.0)	492 (21.3)
Medium	25 339 (41.6)	5481 (40.7)	9348 (41.4)	6517 (42.2)	2967 (42.0)	1026 (44.5)
Low	18 362 (30.2)	4133 (30.7)	6668 (29.5)	4517 (29.3)	2257 (32.0)	787 (34.1)
**Primary infertility**						
No. of women (%)^a^	58 148 (100)	12 853 (22.1)	21 654 (37.2)	14 725 (25.3)	6726 (11.6)	2190 (3.8)
Year of birth, median (IQR), y	1969 (1961-1979)	1968 (1960-1978)	1968 (1961-1977)	1969 (1961-1979)	1971 (1963-1981)	1978 (1966-1985)
Women with infertility, No. (%)	5381 (9.3)	1143 (8.9)	2006 (9.3)	1375 (9.3)	646 (9.6)	211 (9.6)
Age at diagnosis, median (IQR), y	31.3 (27.9-35.1)	31.3 (27.9-35.3)	31.4 (28.0-35.0)	31.0 (27.9-35.1)	30.7 (27.6-35.0)	32.0 (27.7-35.2)
Parental educational level, No. (%)						
High	16 879 (29.0)	3782 (29.4)	6497 (30.0)	4326 (29.4)	1794 (26.7)	480 (21.9)
Medium	24 347 (41.9)	5261 (40.9)	9002 (41.6)	6251 (42.5)	2846 (42.3)	987 (45.1)
Low	16 922 (29.1)	3810 (29.6)	6155 (28.4)	4148 (28.2)	2086 (31.0)	723 (33.0)
**Any infertility**						
No. of women (%)^a^	63 066 (100)	13 877 (22.0)	23 490 (37.3)	16 044 (25.4)	7292 (11.6)	2363 (3.8)
Year of birth, median (IQR), y	1968 (1960-1978)	1967 (1960-1978)	1967 (1960-1977)	1968 (1960-1978)	1971 (1962-1981)	1977 (1966-1985)
Women with infertility, No. (%)	6961 (11.0)	1483 (10.7)	2630 (11.2)	1772 (11.0)	811 (11.1)	265 (11.2)
Age at diagnosis, median (IQR), y	31.9 (28.4-35.7)	32.1 (28.6-35.9)	32.1 (28.6-35.7)	31.8 (28.2-35.8)	31.3 (28.0-35.5)	32.4 (28.4-35.9)
Parental educational level, No. (%)						
High	17 277 (27.4)	3872 (27.9)	6643 (28.3)	4419 (27.5)	1849 (25.4)	494 (20.9)
Medium	26 073 (41.3)	5613 (40.5)	9653 (41.1)	6708 (41.8)	3052 (41.9)	1047 (44.3)
Low	19 716 (31.3)	4392 (31.7)	7194 (30.6)	4917 (30.7)	2391 (32.8)	822 (34.8)

Abbreviations: BMI, body mass index (calculated as weight in kilograms divided by height in meters squared).

^a^ Percentages sum to 100% across columns.

### Childhood BMI and Childbirth

Among 60 864 nulliparous women, associations between childhood BMI trajectories and childbirth differed by age at giving birth but followed similar patterns across age groups. At ages 18 to 24 years, women with an overweight or obesity BMI trajectory in childhood had similar hazards of giving birth as those with the average BMI trajectory in the adjusted analyses (Figure; eTable 1 in Supplement 1). From ages 25 to 29 years to older age groups, women with an overweight or obesity BMI trajectory in childhood had lower hazards of giving birth than women with an average BMI trajectory, and the HRs decreased with increasing age. For example, women with an obesity trajectory had an adjusted HR of 0.78 (95% CI, 0.72-0.86) for giving birth at ages 25 to 29 years and 0.58 (95% CI, 0.52-0.66) for giving birth at ages 30 to 34 years, respectively. By 35 to 45 years, the adjusted HR reached 0.56 (95% CI, 0.46-0.67). Women with an above-average BMI trajectory in childhood had similar hazards of giving birth as women with an average BMI trajectory in childhood at ages 18 to 24 and 25 to 29 years but lower hazards at ages 30 to 34 and 35 to 45 years, with HRs of 0.91 (95% CI, 0.86-0.96) and 0.83 (95% CI, 0.76-0.91), respectively. Women with a below-average BMI trajectory in childhood had similar hazards of giving birth as those with an average BMI trajectory across all age groups.

**Figure.**
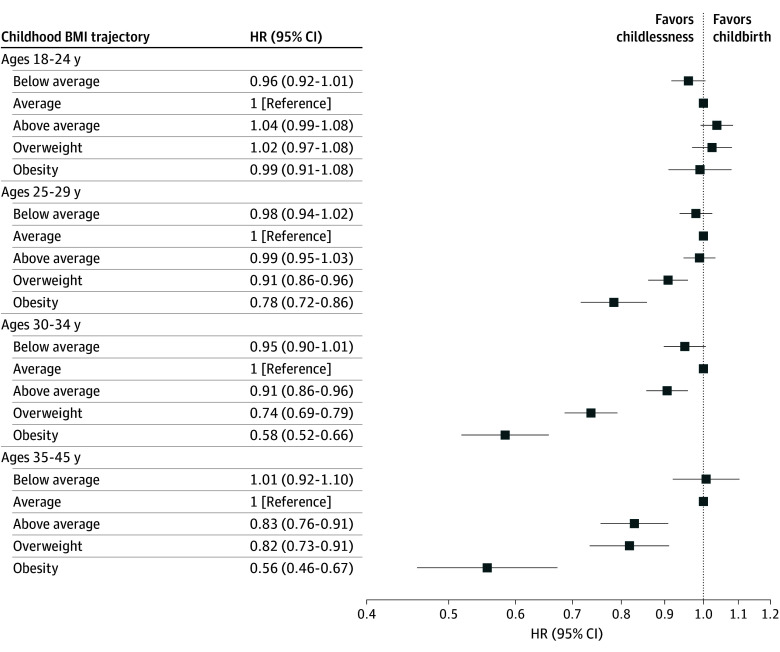
Forest Plot of Hazard Ratios (HRs) for Associations Between Childhood BMI Trajectories and Childbirth by Age Group HRs were estimated using Cox proportional hazard regression models. The models were stratified by 10-year birth cohorts and adjusted for the highest parental educational level achieved. BMI indicates body mass index (calculated as weight in kilograms divided by height in meters squared). Error bars represent 95% CIs.

Secondary analyses using BMI status at ages 7 and 13 years as the exposure showed consistent results (eTable 2 in Supplement 1). Among the 41 485 women who were ever married, associations between childhood BMI trajectories and childbirth were similar to those among all women (eTable 3 in Supplement 1). For women with an obesity BMI trajectory in childhood, the associations were attenuated but remained statistically significant with adjusted HRs of 0.70 (95% CI, 0.61-0.81) and 0.56 (95% CI, 0.43-0.63) for giving birth at ages 30 to 34 and 35 to 45 years, respectively.

### Childhood BMI and Primary Infertility

Among 58 148 nulliparous women, we found that women with an obesity BMI trajectory during childhood had a lower hazard of primary infertility at ages 20 to 45 years than women with an average BMI trajectory, with an adjusted HR of 0.79 (95% CI, 0.68-0.92) (Table 2). No associations were observed between the other childhood BMI trajectories and primary infertility (Table 2). In sensitivity analyses of associations between BMI status at ages 7 and 13 years and primary infertility, similar findings as those in the main analysis were found (eTable 4 in Supplement 1). In the analyses, in which we censored women if they were recorded with IVF treatments unrelated to female infertility, we observed similar results between childhood BMI trajectories and primary infertility as in the main analysis (eTable 5 in Supplement 1). Moreover, when restricting follow-up to ages 30 to 45 years, we observed similar results as those among women with follow-up at ages 20 to 45 years (eTable 6 in Supplement 1).

**Table 2.** Associations Between Childhood BMI Trajectories and Primary Infertility (5381 Cases Among 58 148 Individuals)^a^

Childhood BMI trajectory	HR (95% CI)	
	Unadjusted model^b^	Adjusted model^c^
Below average	0.93 (0.86-1.01)	0.93 (0.86-1.01)
Average	1 [Reference]	1 [Reference]
Above average	0.99 (0.91-1.07)	0.98 (0.91-1.06)
Overweight	0.93 (0.85-1.02)	0.92 (0.84-1.01)
Obesity	0.81 (0.70-0.94)	0.79 (0.68-0.92)

Abbreviations: BMI, body mass index; HR, hazard ratio.

^a^ HRs and 95% CIs were estimated using Cox proportional hazard regression models.

^b^ Stratified by 10-year birth cohorts.

^c^ Stratified by 10-year birth cohorts and adjusted for the highest parental educational level achieved.

### Childhood BMI and Any Infertility

Among 63 066 women regardless of parity, childhood BMI trajectories were not associated with infertility at ages 20 to 45 years. For women with an obesity BMI trajectory, the adjusted HR was 0.97 (95% CI, 0.85-1.10) compared with those with an average BMI trajectory (Table 3). In sensitivity analysis, we found consistent findings when using BMI status at ages 7 and 13 years as in the main analysis (eTable 7 in Supplement 1). Moreover, in the analyses where women were censored if they were recorded with IVF treatment unrelated to female infertility, we found no associations between childhood BMI trajectories and any infertility (eTable 8 in Supplement 1).

**Table 3.** Associations Between Childhood BMI Trajectories and Any Infertility (6961 Cases Among 63 066 Individuals)^a^

Childhood BMI trajectory	HR (95% CI)	
	Unadjusted model^b^	Adjusted model^c^
Below average	0.95 (0.89-1.02)	0.95 (0.89-1.03)
Average	1 [Reference]	1 [Reference]
Above average	0.98 (0.92-1.05)	0.99 (0.92-1.06)
Overweight	0.98 (0.90-1.06)	0.98 (0.91-1.07)
Obesity	0.95 (0.84-1.08)	0.97 (0.85-1.10)

Abbreviations: BMI, body mass index; HR, hazard ratio.

^a^ HRs and 95% CIs were estimated using Cox proportional hazard regression models.

^b^ Stratified by 10-year birth cohorts.

^c^ Stratified by 10-year birth cohorts and adjusted for the highest parental educational level achieved.

## Discussion

Our large cohort and its detailed follow-up enabled us to examine age-specific associations between childhood BMI and childbirth, an approach not used in prior studies. In our study, women with above-average, overweight, and obesity BMI trajectories in childhood had lower hazards of childbirth than women with an average BMI trajectory, supporting our hypothesis. Notably, we observed associations emerging as early as ages 25 to 29 years, well before most women typically seek evaluation or treatment for infertility. By ages 30 to 34 and 35 to 45 years, when infertility care is more commonly sought, these associations were even more pronounced. We found no association among women with a below-average BMI trajectory. Contrary to our hypothesis, trajectories of BMI across childhood were not associated with primary or any infertility in adulthood, although women with the obesity trajectory had a lower hazard of primary infertility.

Our findings of lower hazards of childbirth among women with an above-average BMI during childhood are consistent with 2 previous well-conducted studies, although both were smaller in scale and did not examine age-specific associations.^10,11^ In a multiethnic cohort of US women, self-reported obesity in high school was associated with higher risk of self-reported lifetime nulliparity.^10^ Likewise, among Finnish women with measured childhood weight and height, overweight and obesity at ages 11 to 15 years were associated with higher risks of childlessness from registry follow-up.^11^ Taken together, these findings suggest that the influence of elevated childhood BMI on reproductive outcomes may begin early in life. By the time women reach their 30s, when fertility difficulties often become apparent, these effects may already be established, underscoring the need for preventive efforts focused on healthy growth and weight management well before the reproductive age.

The lower hazard of childbirth among women with overweight and obesity throughout childhood may result from physiological, social, or combined mechanisms. In the sensitivity analysis among ever-married women in our study, associations between childhood BMI trajectories of overweight and obesity and childbirth were similar to those in the full cohort, although attenuated but still statistically significant. While this finding should be replicated in cohorts with more detailed information on factors influencing reproductive decisions, it suggests that difficulties in forming partnerships alone does not fully explain the observed associations. This finding is notable given prior evidence showing that women with obesity in childhood have lower odds of marriage and cohabitation than women with normal weight.^26,27^ Biological mechanisms are also likely to contribute. Excess adiposity in childhood and adolescence may negatively impact the hypothalamic pituitary ovarian axis, oocyte quality, and the endometrium as observed in adult women,^28^ but this possibility is underinvestigated.

Overall, childhood BMI trajectories were not associated with diagnoses of primary or any infertility. However, adjusted models showed a lower hazard of primary infertility among women with the obesity trajectory. We speculate that this inverse association likely reflects differences in access to fertility care rather than a biological mechanism. In Denmark, infertility investigations typically begin in primary care, and national guidelines restrict access to IVF treatment for women with a BMI above 30 if younger than 30 years or a BMI of 35 if older than 30 years due to greater risks of pregnancy complications.^29^ Women who exceed these BMI thresholds may never be referred for treatment. Because obesity in childhood often persists into adulthood,^30^ restricted access to hospital-based fertility services can plausibly explain the inverse association between childhood obesity and hospital-based infertility diagnoses. Therefore, this finding should be interpreted with great caution. Comparisons with previous studies are challenging because infertility is assessed in many ways. The 4 prior studies^11,12,13,14^ used self-reported measures ranging from difficulties in conceiving within 12 months to seeking medical help for infertility, and these studies may capture a broader spectrum of cases than hospital-based diagnoses. Despite these methodological differences, 2 studies reported results consistent with ours, showing no associations between overweight or obesity in childhood and infertility,^11,12^ whereas 2 other studies observed positive associations between obesity before, but not after, age 12 years.^13,14^

### Strengths and Limitations

This study has several strengths. We used data from a large population-based cohort that includes nearly every child attending school in Copenhagen to create a strong study design. Both private and public schools offered legally mandated health examinations, which reduces the risk of selection bias by SEP. Moreover, the BMIs were calculated from measured weight and height performed by trained personnel, which increases the precision and limits the risk of potential misclassification. We captured all childbirths and hospital-based diagnoses of infertility during the study period, which included information from 1 nationwide cohort and 3 national health registers. This approach minimizes the risk of undetected childbirths and infertility diagnoses.

This study also has some limitations. Despite universal health care and free initial IVF treatment in Denmark, women with lower educational attainment are less likely to seek medical help for infertility.^31^ Thus, infertility among women with lower SEP may be underestimated. Although we accounted for parental educational level, we lacked information on other potential confounders. Given the early life exposure, many later life factors act as mediators rather than confounders. However, residual and unmeasured confounding cannot be excluded. Another limitation is that we were unable to obtain information on BMI in adulthood, which precluded the examination of the importance of excess adiposity at ages when trying to conceive. Nonetheless, because women tend to discover difficulties with conceiving at ages close to when IVF treatment is no longer offered, it is essential that early life indicators of adverse reproductive outcomes are identified.

## Conclusions

In this cohort study of Danish women, we found that those with an overweight or obesity BMI trajectory in childhood were less likely to have children than women with an average BMI trajectory. The association was already evident at ages 25 to 29 years and, notably, became more apparent after age 30 years, which is when many women start trying to conceive. In contrast, childhood BMI trajectories were generally not associated with primary or any infertility. Our results emphasize that women’s reproductive health needs to be addressed many years before women wish to become pregnant and suggest that childhood may be an important period for supporting women’s later reproductive health.

## Notes

Supplement 1.
eMethods.
**eFigure.** Flowchart of Included and Excluded Women From the Copenhagen School Health Records Register **eTable 1.** Associations Between Childhood BMI Trajectories and Childbirth **eTable 2.** Estimates From Associations Between Childhood BMI Categories and Childbirth **eTable 3.** Estimates From Associations Between Childhood BMI Trajectories and Childbirth – Among Women Who Were Married **eTable 4.** Associations Between Childhood BMI Categories and Primary Infertility **eTable 5.** Associations Between Childhood BMI Trajectories and Primary Infertility – With Censoring on Women Who Were Registered With Male Infertility, Same Sex Partnership, or Single Status **eTable 6.** Estimates From Associations Between Childhood BMI Trajectories and Primary Infertility – Among Women Aged 30 to 45 Years **eTable 7.** Associations Between Childhood BMI Categories and Any Infertility **eTable 8.** Associations Between Childhood BMI Trajectories and Any Infertility – With Censoring on Women Who Were Registered With Male Infertility, Same Sex Partnership, or Single Status

Supplement 2.
Data Sharing Statement
