# Supplementary material for: Early Life BMI Trajectories and Associations With Childbirth and Infertility Among Women
Source: JAMA Netw Open. 2026 Aug 4;9(8):e2626893. doi: 10.1001/jamanetworkopen.2026.26893 (PMC13439426; doi:10.1001/jamanetworkopen.2026.26893)
Supplement: Supplementary file 1 [file jamanetwopen-e2626893-s001.pdf]

Supplemental Online Content

Pedersen DC, Aarestrup J, Bjerregaard LG, et al. Early life BMI trajectories and associations with childbirth and infertility among women. *JAMA Netw Open*. 2026;9(8):e2626893. doi:10.1001/jamanetworkopen.2026.26893.

eMethods
eFigure 1. Flowchart of Included and Excluded Women From the Copenhagen School Health Records Register
eTable 1. Associations Between Childhood BMI Trajectories and Childbirth
eTable 2. Estimates From Associations Between Childhood BMI Categories and Childbirth
eTable 3. Estimates From Associations Between Childhood BMI Trajectories and Childbirth – Among Women Who Were Married
eTable 4. Associations Between Childhood BMI Categories and Primary Infertility
eTable 5. Associations Between Childhood BMI Trajectories and Primary Infertility – With Censoring on Women Who Were Registered With Male Infertility, Same Sex Partnership, or Single Status
eTable 6. Estimates From Associations Between Childhood BMI Trajectories and Primary Infertility – Among Women Aged 30 to 45 Years
eTable 7. Associations Between Childhood BMI Categories and Any Infertility
eTable 8. Associations Between Childhood BMI Trajectories and Any Infertility – With Censoring on Women Who Were Registered With Male Infertility, Same Sex Partnership, or Single Status

This supplemental material has been provided by the authors to give readers additional information about their work.

© 2026 Pedersen DC et al. JAMA Network Open.

eMethods

Latent class trajectory modelling

We used latent class trajectory models to identify trajectories of body mass index (BMI) across ages 6 to 15 years. All girls in the Copenhagen School Health Records Register (CSHRR) with a minimum of two BMI values were included in the models. The trajectories were modelled using the LCMM package (version 1.8.1) in R (version 3.6.1). Age was used as the timescale, and modelled with natural splines with three knots placed at ages 8, 10, and 12 years. These knots were selected a priori and chosen to reflect natural changes in children's growth. The trajectories were adjusted for 5-year birth cohorts. Models with different random effect structures were tested. We used the "Gridsearch" function in R to ensure that the model converged at the global maximum.

Models with one to eight classes were evaluated. We identified the optimal model using a combination of several fit indices, including the proportion of individuals in each trajectory, Bayesian Information Criterion, the average posterior probability, the relative entropy, and the odds of correct classification.[1] Moreover, we used visual inspection of the models. A model with no random intercept and slope and five distinct trajectories were evaluated as the most optimal model. Further details can be found in Jensen et al., 2023.[2]

Supplementary references

1. Lennon H, Kelly S, Sperrin M, et al. Framework to construct and interpret latent class trajectory modelling. *BMJ Open* 2018;8(7):e020683.
2. Jensen BW, Aarestrup J, Blond K, et al. Childhood body mass index trajectories, adult-onset type 2 diabetes, and obesity-related cancers. *J Natl Cancer Inst*. 2023;115:43-51. https://doi.org/10.1093/jnci/djac192

Study populations

Of the starting population of 84,508 women born 1950 to 1989, we generated three analytic cohorts for the three main outcomes 1) childbirth, 2) primary infertility and 2) any infertility.

Childbirth

For the analyses on childbirth, we initiated follow-up on January 1, 1977, or age 18 years whichever came last. Women were excluded if they had an oophorectomy, a hysterectomy or a tubal ligation

© 2026 Pedersen DC et al. JAMA Network Open.

before age 18 years (n=9), if they gave birth prior to study entrance (n=9,452; 11.2%). Moreover, we excluded women with missing information on childhood BMI trajectories (n=2,935; 3.5%) and parental education (n=9,454; 11.2%) (eFigure 1). Follow-up ended on the date of childbirth, age 45 years, on the date of having a procedure of oophorectomy, a hysterectomy or a tubal ligation, or other loss to follow-up, December 31, 2022, which ever came first.

In sensitivity analyses among women with information on marriage before age 45 years, 49,120 (80.7%) women out of 60,864 were included.

Primary infertility

For the analyses on primary infertility, we initiated follow-up on January 1, 1977, or age 20 years whichever came last. Women were excluded if they had an oophorectomy, a hysterectomy or a tubal ligation before age 20 years (n=16), if they were diagnosed with infertility prior to study entrance (n=70; 0.1%), or if they had given birth prior to study entrance (n=12,347; 14.6%). Moreover, we excluded women with missing information on childhood BMI trajectories (n=2,730; 3.2%) and parental education (n=9,155; 10.8%) (eFigure 1). Follow-up ended on the date of diagnosis of infertility, date of childbirth, age 45 years, on the date of having a procedure of oophorectomy, a hysterectomy or a tubal ligation, or other loss to follow-up, December 31, 2022, which ever came first.

Any infertility

For the analyses on any infertility, we initiated follow-up on January 1, 1977, or age 20 years whichever came last. Women were excluded if they had an oophorectomy, a hysterectomy or a tubal ligation before age 20 years (n=16), if they were diagnosed with infertility prior to study entrance (n=70; 0.1%). Moreover, we excluded women with missing information on childhood BMI trajectories (n=3,014; 3.6%) and parental education (n=16,300; 19.3%) (eFigure 1). Follow-up ended on the date of diagnosis of infertility, age 45 years, on the date of having a procedure of oophorectomy, a hysterectomy or a tubal ligation, or other loss to follow-up, December 31, 2022, which ever came first.

© 2026 Pedersen DC et al. JAMA Network Open.

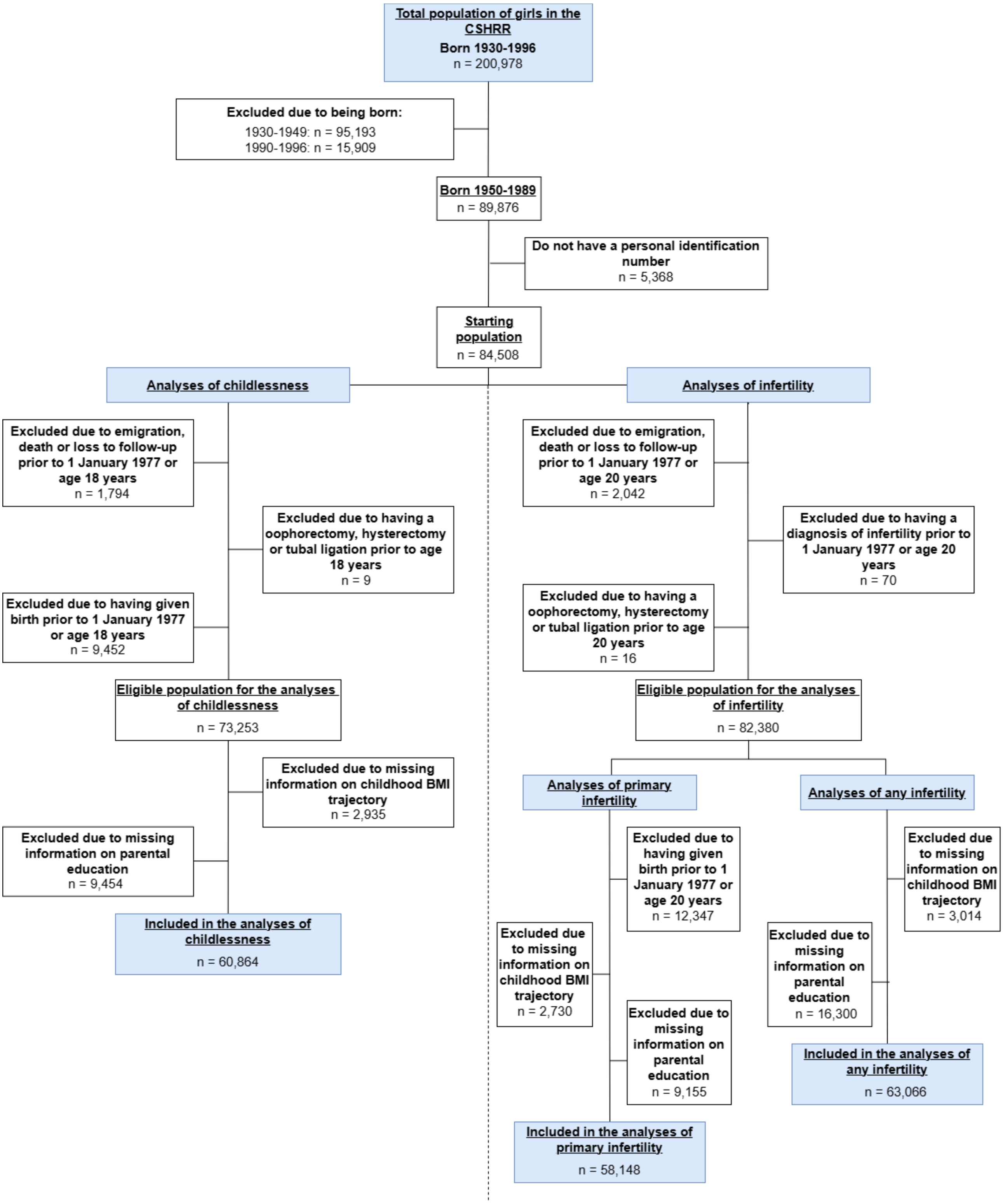

eFigure 1. Flowchart of included and excluded women from the Copenhagen School Health Records Register
Abbreviations: BMI, body mass index; CSHRR, Copenhagen School Health Records Register

© 2026 Pedersen DC et al. JAMA Network Open.

Childbirth analyses

eTable 1. Associations between childhood BMI trajectories and childbirth[a]

	Unadjusted model[b] N = 60,864 (events = 47,669) HR (95% CI) Age-group (years)				Adjusted model[c] N = 60,864 (events = 47,669) HR (95% CI) Age-group (years)			
Childhood BMI trajectory	18-24	25-29	30-34	35-45	18-24	25-29	30-34	35-45
Below-average	0.97 (0.93-1.02)	0.98 (0.94-1.03)	0.95 (0.89-1.00)	1.00 (0.91-1.09)	0.96 (0.92-1.01)	0.98 (0.94-1.02)	0.95 (0.90-1.01)	1.01 (0.92-1.10)
Average	Ref.	Ref.	Ref.	Ref.	Ref.	Ref.	Ref.	Ref.
Above-average	1.05 (1.00-1.09)	0.99 (0.95-1.04)	0.90 (0.85-0.95)	0.82 (0.75-0.90)	1.04 (0.99-1.08)	0.99 (0.95-1.03)	0.91 (0.86-0.96)	0.83 (0.76-0.91)
Overweight	1.09 (1.03-1.15)	0.93 (0.88-0.98)	0.73 (0.68-0.78)	0.79 (0.71-0.88)	1.02 (0.97-1.08)	0.91 (0.86-0.96)	0.74 (0.69-0.79)	0.82 (0.73-0.91)
Obesity	1.14 (1.04-1.24)	0.82 (0.75-0.90)	0.56 (0.50-0.64)	0.52 (0.43-0.63)	0.99 (0.91-1.08)	0.78 (0.72-0.86)	0.58 (0.52-0.66)	0.56 (0.46-0.67)

[a] Hazard ratios and 95% confidence intervals were estimated using Cox proportional hazard regression models

[b] Stratified by 10-year birth cohorts

[c] Stratified by 10-year birth cohorts and adjusted for highest parental educational level achieved

BMI, body mass index; CI, confidence intervals; HR, hazard ratio

© 2026 Pedersen DC et al. JAMA Network Open.

eTable 2. Estimates from associations between childhood BMI categories and childbirth[a]

	Unadjusted model[b]				Adjusted model[c]			
	HR (95% CI)				HR (95% CI)			
	Age-group (years)				Age-group (years)			
Childhood BMI category	18-24	25-29	30-34	35-45	18-24	25-29	30-34	35-45
Age 7 years	N = 56,919 (events = 44,717)				N = 56,929 (events = 44,717)			
Underweight	0.93 (0.86-1.01)	1.00 (0.93-1.08)	1.04 (0.94-1.14)	0.92 (0.78-1.08)	0.91 (0.84-0.98)	1.00 (0.93-1.07)	1.04 (0.95-1.14)	0.94 (0.80-1.10)
Normal weight	Ref.	Ref.	Ref.	Ref.	Ref.	Ref.	Ref.	Ref.
Overweight	0.95 (0.89-1.01)	0.91 (0.86-0.97)	0.78 (0.73-0.85)	0.83 (0.74-0.93)	0.91 (0.86-0.97)	0.90 (0.85-0.95)	0.79 (0.73-0.86)	0.85 (0.75-0.95)
Obesity	0.90 (0.80-1.01)	0.86 (0.77-0.96)	0.72 (0.62-0.82)	0.60 (0.47-0.76)	0.81 (0.72-0.92)	0.83 (0.75-0.93)	0.73 (0.64-0.84)	0.63 (0.49-0.80)
Age 13 years	N = 45,131 (events = 35,254)				N = 45,131 (events = 35,254)			
Underweight	0.95 (0.88-1.03)	0.95 (0.88-1.03)	1.03 (0.93-1.13)	1.03 (0.88-1.2)	0.93 (0.86-1.01)	0.94 (0.87-1.02)	1.03 (0.93-1.14)	1.04 (0.89-1.22)
Normal weight	Ref.	Ref.	Ref.	Ref.	Ref.	Ref.	Ref.	Ref.
Overweight	1.10 (1.03-1.18)	0.91 (0.85-0.98)	0.73 (0.67-0.81)	0.82 (0.71-0.94)	1.03 (0.96-1.10)	0.89 (0.83-0.96)	0.75 (0.68-0.82)	0.84 (0.73-0.97)
Obesity	1.15 (1.01-1.3)	0.72 (0.63-0.84)	0.52 (0.43-0.62)	0.55 (0.41-0.74)	1.00 (0.88-1.13)	0.70 (0.60-0.81)	0.53 (0.44-0.65)	0.59 (0.44-0.80)

[a] Hazard ratios and 95% confidence intervals were estimated using Cox proportional hazard regression models

[b] Stratified by 10-year birth cohorts

[c] Stratified by 10-year birth cohorts and adjusted for highest parental educational level achieved

BMI, body mass index; CI, confidence intervals; HR, hazard ratio

© 2026 Pedersen DC et al. JAMA Network Open.

eTable 3. Estimates from associations between childhood BMI trajectories and childbirth – among women who were married[a]

	Unadjusted model[b] N = 41,485 (events = 36,243)				Adjusted model[c] N = 41,485 (events = 36,243)			
	HR (95% CI)				HR (95% CI)			
	Age-group (years)				Age-group (years)			
Childhood BMI trajectory	18-24	25-29	30-34	35-45	18-24	25-29	30-34	35-45
Below-average	0.99 (0.94-1.05)	0.99 (0.94-1.04)	0.95 (0.89-1.01)	1.04 (0.93-1.16)	0.98 (0.93-1.04)	0.99 (0.94-1.04)	0.95 (0.89-1.02)	1.05 (0.94-1.17)
Average	Ref.	Ref.	Ref.	Ref.	Ref.	Ref.	Ref.	Ref.
Above-average	1.07 (1.02-1.12)	1.02 (0.97-1.07)	0.97 (0.91-1.03)	0.81 (0.72-0.91)	1.07 (1.01-1.12)	1.01 (0.97-1.06)	0.97 (0.91-1.04)	0.82 (0.73-0.92)
Overweight	1.15 (1.08-1.22)	0.96 (0.9-1.02)	0.75 (0.69-0.82)	0.79 (0.69-0.90)	1.08 (1.01-1.15)	0.94 (0.88-1.00)	0.77 (0.71-0.84)	0.82 (0.72-0.94)
Obesity	1.29 (1.17-1.42)	0.97 (0.87-1.07)	0.67 (0.58-0.78)	0.53 (0.41-0.68)	1.13 (1.02-1.24)	0.93 (0.84-1.03)	0.70 (0.61-0.81)	0.56 (0.43-0.73)

[a] Hazard ratios and 95% confidence intervals were estimated using Cox proportional hazard regression models

[b] Stratified by 10-year birth cohorts

[c] Stratified by 10-year birth cohorts and adjusted for highest parental educational level achieved

BMI, body mass index; CI, confidence intervals; HR, hazard ratio

© 2026 Pedersen DC et al. JAMA Network Open.

Primary infertility analyses

eTable 4. Associations between childhood BMI categories and primary infertility[a]

			Unadjusted model[b]	Adjusted model[c]
Childhood BMI categories	Total	*n*	HR (95% CI)	HR (95% CI)
Age 7 years				
Underweight	2,349	197	0.83 (0.72-0.96)	0.83 (0.72-0.96)
Normal weight	42,261	4,364	Ref.	Ref.
Overweight	3,719	421	0.94 (0.85-1.04)	0.94 (0.85-1.04)
Obesity	1,070	104	0.76 (0.62-0.92)	0.74 (0.61-0.90)
Age 13 years				
Underweight	2,203	166	0.78 (0.66-0.91)	0.77 (0.66-0.90)
Normal weight	33,438	3,184	Ref.	Ref.
Overweight	2,892	293	0.92 (0.81-1.03)	0.90 (0.80-1.02)
Obesity	758	84	0.86 (0.69-1.07)	0.84 (0.67-1.04)

[a] Hazard ratios and 95% confidence intervals were estimated using Cox proportional hazard regression models

[b] Stratified by 10-year birth cohorts

[c] Stratified by 10-year birth cohorts and adjusted for highest parental educational level achieved

BMI, body mass index; CI, confidence intervals; HR, hazard ratio

eTable 5. Associations between childhood BMI trajectories and primary infertility – with censoring on women who were registered with male infertility, same sex partnership, or single status[a]

			Unadjusted model[b]	Adjusted model[c]
Childhood BMI trajectory	Total	*n*	HR (95% CI)	HR (95% CI)
Below-average			0.97 (0.88-1.06)	0.96 (0.88-1.06)
Average			Ref.	Ref.
Above-average	58,148	4,261	1.03 (0.95-1.13)	1.03 (0.94-1.12)
Overweight			0.95 (0.85-1.06)	0.94 (0.84-1.04)
Obesity			0.86 (0.72-1.01)	0.83 (0.70-0.98)

[a] Hazard ratios and 95% confidence intervals were estimated using Cox proportional hazard regression models

[b] Stratified by 10-year birth cohorts

[c] Stratified by 10-year birth cohorts and adjusted for highest parental educational level achieved

BMI, body mass index; CI, confidence intervals; HR, hazard ratio

© 2026 Pedersen DC et al. JAMA Network Open.

eTable 6. Associations between childhood BMI trajectories and primary infertility – among women aged 30 to 45 years[a]

			Unadjusted model[b]	Adjusted model[c]
Childhood BMI trajectory	Total	*n*	HR (95% CI)	HR (95% CI)
Below-average	24,836	3,162	0.91 (0.82-1.02)	0.92 (0.82-1.02)
Average			Ref.	Ref.
Above-average			0.94 (0.85-1.04)	0.94 (0.85-1.05)
Overweight			0.81 (0.72-0.92)	0.82 (0.72-0.93)
Obesity			0.74 (0.61-0.89)	0.76 (0.63-0.92)

[a] Hazard ratios and 95% confidence intervals were estimated using Cox proportional hazard regression models

[b] Stratified by 10-year birth cohorts

[c] Stratified by 10-year birth cohorts and adjusted for highest parental educational level achieved

BMI, body mass index; CI, confidence intervals; HR, hazard ratio

© 2026 Pedersen DC et al. JAMA Network Open.

Any infertility analyses

eTable 7. Associations between childhood BMI categories and any infertility[a]

Childhood BMI categories	Total	*n*	Unadjusted model[b] HR (95% CI)	Adjusted model[c] HR (95% CI)
Age 7 years				
Underweight	2,500	250	0.83 (0.73-0.94)	0.83 (0.73-0.94)
Normal weight	44,958	5,659	Ref.	Ref.
Overweight	3,912	520	1.02 (0.93-1.11)	1.02 (0.93-1.12)
Obesity	1,102	134	0.92 (0.77-1.09)	0.93 (0.78-1.10)
Age 13 years				
Underweight	2,378	218	0.82 (0.72-0.94)	0.82 (0.72-0.94)
Normal weight	36,057	4,170	Ref.	Ref.
Overweight	3,146	362	0.96 (0.86-1.07)	0.97 (0.87-1.08)
Obesity	817	96	0.95 (0.78-1.17)	0.97 (0.79-1.19)

[a] Hazard ratios and 95% confidence intervals were estimated using Cox proportional hazard regression models

[b] Stratified by 10-year birth cohorts

[c] Stratified by 10-year birth cohorts and adjusted for highest parental educational level achieved

BMI, body mass index; CI, confidence intervals; HR, hazard ratio

eTable 8. Associations between childhood BMI trajectories and any infertility – with censoring on women who were registered with male infertility, same sex partnership, or single status[a]

Childhood BMI trajectory	Total	*n*	Unadjusted model[b] HR (95% CI)	Adjusted model[c] HR (95% CI)
Below-average			0.98 (0.91-1.07)	0.99 (0.91-1.07)
Average			Ref.	Ref.
Above-average	63,066	5,566	1.01 (0.94-1.09)	1.01 (0.94-1.09)
Overweight			1.00 (0.91-1.10)	1.00 (0.92-1.10)
Obesity			0.98 (0.84-1.13)	0.99 (0.85-1.15)

[a] Hazard ratios and 95% confidence intervals were estimated using Cox proportional hazard regression models

[b] Stratified by 10-year birth cohorts

[c] Stratified by 10-year birth cohorts and adjusted for highest parental educational level achieved

BMI, body mass index; CI, confidence intervals; HR, hazard ratio

© 2026 Pedersen DC et al. JAMA Network Open.
