# Supplementary material for: Early Life BMI Trajectories and Associations With Childbirth and Infertility Among Women
Source: JAMA Netw Open. 2026 Aug 4;9(8):e2626893. doi: 10.1001/jamanetworkopen.2026.26893 (PMC13439426; doi:10.1001/jamanetworkopen.2026.26893)
Supplement: Supplementary file 2 [file jamanetwopen-e2626893-s002.pdf]

Data Sharing Statement

Pedersen. Early Life BMI Trajectories and Associations With Childbirth and Infertility Among Women. *JAMA Netw Open*. Published August 04, 2026.
doi:10.1001/jamanetworkopen.2026.26893

Data
Data available: No

Additional Information
Explanation for why data not available: The dataset used in this study is not publicly available due to being pseudo-anonymized, but access can be granted pending approval from the steering committees for the Copenhagen School Health Records Register and the Danish Infertility Cohort following standardized access procedures. The statistical code used for the present analyses can be made available upon request to the last author.
